# Impaired Inter-Hemispheric Functional Connectivity during Resting State in Female Patients with Migraine

**DOI:** 10.3390/brainsci12111505

**Published:** 2022-11-06

**Authors:** Yanan Zhang, Ni Liu, Zhenjia Wang, Junlian Liu, Mengmeng Ren, Yueying Hong, Xuanzhi Luo, Huilin Liu, Jianwei Huo, Zhenchang Wang

**Affiliations:** 1 Department of Radiology, Beijing Friendship Hospital, Capital Medical University, Beijing 100050, China; 2 Department of Radiology, Beijing Hospital of Traditional Chinese Medicine, Capital Medical University, Beijing 100010, China; 3 Acupuncture and Moxibustion Department, Beijing Hospital of Traditional Chinese Medicine, Capital Medical University, Beijing 100010, China

**Keywords:** migraine, female, functional magnetic resonance imaging, functional connectivity, voxel-mirrored homotopic connectivity

## Abstract

The application of voxel-mirrored homotopic connectivity (VMHC) analysis to study the central mechanism of migraine has been limited. Furthermore, little is known about inter-hemispheric functional connectivity (FC) alterations during resting state in female patients with migraine. This study aimed to investigate potential interictal VMHC impairments in migraine without aura (MwoA) patients and the relationship between connectivity alterations and clinical parameters. Resting-state functional magnetic resonance imaging data and clinical information were acquired from 43 female MwoA patients and 43 matched healthy controls. VMHC analysis was used to compare differences between these two groups, and brain regions showing significant differences were chosen as a mask to perform a seed-based FC group comparison. Subsequent correlation analysis was conducted to explore the relationship between abnormal inter-hemispheric FC and clinical data. Compared with healthy controls, female MwoA patients revealed significantly decreased VMHC in the bilateral cerebellum; cuneus; and lingual, middle occipital, precentral and postcentral gyri. Seed-based FC analysis indicated disrupted intrinsic connectivity in the cerebellum, and default mode, visual and sensorimotor network. These VMHC and FC abnormalities were negatively correlated with clinical indexes including duration of disease, migraine days and visual analogue scale. These inter-hemispheric FC impairments and correlations between abnormal VMHC and FC and clinical scores may improve our understanding of the central mechanism of female-specific migraine.

## 1. Introduction

Migraine is a general primary headache that can be divided into two main types: migraine with aura and migraine without aura (MwoA) [1], which accounts for approximately 80% of all migraines. Migraines occur three times more frequently in women than in men [2]; furthermore, headache attacks in females are more severe, more frequent, longer lasting, and more likely to become chronic [3,4]. Previous studies have shown the incidence of migraine in women is associated with fluctuations in ovarian steroid hormone levels [5], especially estrogen [6]. Cyclical changes in sex hormones can alter the function of neurons in the brain, increasing susceptibility for migraine attacks [5], which is supported by neuroimaging evidence [6,7]. Maleki, et al. (2012) speculated that sex differences in migraine involved brain function and structure; they found that female patients had thicker cortices in the posterior insula and precuneus and greater activation of emotional circuitry compared with both male patients and healthy controls (HCs) [8]. Another study on gender-related differences also suggested that migraine might have a greater influence on females in the resting functional networks compared to males [9].

In recent years, the resting-state functional magnetic resonance imaging (rs-fMRI) has become an essential tool to explore the central mechanism underlying migraine. An increasing number of rs-fMRI studies demonstrate that abnormal pain-related brain regions, networks, and topological characteristics may be involved in the central mechanism of migraine [10,11,12,13]. Functional connectivity (FC) is the most commonly used analytical method in rs-fMRI, and it examines the temporal correlation in blood-oxygen-level-dependent signal changes between different regions of the brain. FC can describe how various brain regions interact when the brain is in resting or active state [14]. A recent review reported that migraineurs had abnormal FC or seed areas in the salience network, somatosensory network, default mode network (DMN), pons, and thalamus during the attack relative to no-headache controls. Furthermore, migraine patients have also exhibited aberrant FC or seed areas including the periaqueductal gray (PAG), fronto-parietal-network, and salience network during the interictal phase relative to HCs [10]. Related research discovered FC alterations associated with female migraine in several networks including the PAG, DMN, visual network, sensorimotor network (SMN), and limbic system [8,15,16]. Existing studies have mainly analyzed the FC between regions of interest (ROIs) and the whole brain. However, inter-hemispheric FC changes between the bilateral hemispheres have not yet been clearly studied.

The voxel-mirrored homotopic connectivity (VMHC) method is a novel rs-fMRI analysis index that evaluates FC strength between the bilateral hemispheres; it primarily reflects the inter-hemispheric information of communication and coordination function through high synchronization of spontaneous activity in symmetrical regions of the left and right hemispheres [17]. It is widely used in a variety of studies on neurological disorders [18,19,20,21]. A VMHC analysis between 21 MwoA patients and 21 HCs demonstrated decreased VMHC value in the anterior cingulate cortex (ACC) in patients relative to controls. Meanwhile, reduced fractional anisotropy was observed in the genu of the corpus callosum in MwoA patients. Further analysis revealed a significant positive correlation between the structural and functional changes, indicating that changes in the corpus callosum may modulate inter-hemispheric FC [22]. Additionally, the VMHC of the bilateral middle frontal gyrus, precuneus, ACC and postcentral gyrus has been shown to be significantly increased in patients with menstrual migraine compared with HCs, and the VMHC value of bilateral ACC correlated positively with headache intensity [23]. A recent rs-fMRI study suggested that patients with migraine exhibited higher VMHC and altered FC in the bilateral thalamus and other brain areas. Moreover, these abnormal FC values were found to be negatively correlated with visual analogue scale (VAS) scores or headache attack durations. These abnormalities may be related to the neural mechanism of migraine [20]. Unfortunately, existing research results have been inconsistent, and relatively little is known about the alterations in the inter-hemispheric FC during the resting state in female MwoA patients; therefore, more research is needed to clarify these alterations.

We propose the hypothesis that female MwoA patients exhibit VMHC alterations in some pain-related brain regions relative to HCs. In this study, we collected rs-fMRI data and clinical parameters in patients and HCs. First, we calculated VMHC between two groups. Then, we conducted seed-based FC analysis in brain regions with significant VMHC alterations. Finally, we investigated the relationship between abnormal imaging data and clinical scores.

## 2. Material and Methods

### 2.1. Study Design

**Figure 1:**
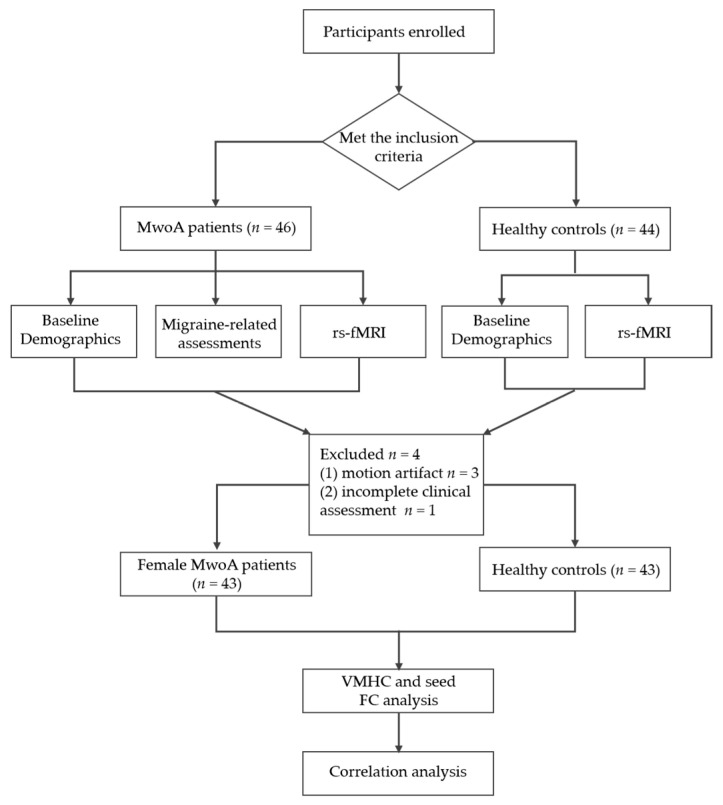
Study design flow chart.

This study was designed as a cross-sectional study to observe VMHC alterations and seed-based FC changes in female MwoA patients compared to HCs. The screening, enrollment, exclusion, and assessment process is outlined in the flow chart (Figure 1). The sample size was calculated according to the estimation of statistical power in fMRI. At least 24 participants were required to achieve 80% power at the single voxel level for typical activations and multiple comparison correction [24,25].

### 2.2. Participants

The present study was approved by the Ethics Committee of Beijing Hospital of Traditional Chinese Medicine (2021BL02-055-02). The study was performed according to the principles of the Declaration of Helsinki (as revised in 2013). Informed consent was obtained in writing from all participants prior to participation in the study.

Each MwoA patient was prospectively recruited from the outpatient department of the Acupuncture and Moxibustion clinic in our hospital between February 2017 and March 2022. The inclusion criteria for patients were as follows: (1) met the diagnostic criteria of MwoA according to the International Classification of Headache Disorders III (beta version) [1]; (2) female; (3) 18–65 years of age; (4) right-handed; (5) at least one year of migraine duration; and (6) greater than twice per month migraine attack frequency. Additionally, HCs were recruited from advertisements in the community and online. The inclusion criteria for HCs were as follows: female, no significant difference from patients in age and education level, right-handed, good health, absence of headache, and no obvious whole-brain structural abnormalities from MRI results.

Participants were eliminated if they satisfied the following exclusion criteria: (1) other secondary types of headache, such as rhinogenic headache [26]; (2) any other neurological, psychiatric and relatively severe life-threatening diseases (e.g., cardiovascular disorders, and acute infection); (3) pregnancy or lactation; (4) use of prophylactic treatment for migraine in the three months prior to the study; (5) MRI exam contraindications; (6) any abnormal or asymmetric structural abnormalities, except for Fazekas grade 1 lesion in the white matter (according to the Fazekas scale by Fazekas et al. (1987) [27]); (7) alcohol or drug abuse.

The basic information collected for each subject included sex, age, years of education, and disease duration. Additional information was recorded for each MwoA patient, including headache location, frequency, intensity, migraine days, concomitant symptoms, VAS assessment, Beck Anxiety Inventory (BAI), and Beck Depression Inventory (BDI). The VAS indicates the level of pain that the subject experienced based on a 10 cm line scale, where 0 = no pain and 10 = greatest pain imaginable. The BAI is a 21-item assessment that evaluates the severity of anxiety in patients based on the following scoring system: A score of 15–25 indicates mild anxiety, 26–35 indicates moderate anxiety, and 36 or more indicates severe anxiety. The BDI is a 21-question assessment that determines the degree of depression based on the following scoring system: A score of 10 or less indicates no depression, 10–15 indicates a mild mood disorder that does not meet the criteria for depression, 15–25 indicates mild depression, and 25 or more indicates serious depression.

### 2.3. MRI Data Acquisition

All imaging data were acquired using a 3.0 Tesla scanner (Magnetom Skyra, Siemens, Erlangen, Germany) at the Department of Radiology in our hospital. The MRI was scheduled during the interictal migraine period such that the patients did not suffer from a migraine attack at least 72 h before the scan. The earmuff and sponge cushion were used to minimize noise exposure and head motion. The subjects were required to close their eyes, remain awake and relaxed, and avoid systematic thinking.

First, T1-weighted whole brain images were obtained using the following parameters: repetition time (TR) = 2300 ms; echo time (TE) = 2.32 ms; slice thickness = 0.9 mm, dist factor = 50%; slices = 192; flip angle = 8°; field of view (FOV) = 240×240 mm^2^; matrix size = 256 × 256. Next, rs-fMRI was performed using an echo-planar imaging sequence and the following scan parameters: TR = 3000 ms; TE = 30 ms; voxel size = 2.3 × 2.3 × 3.0 mm^3^, dist factor = 25%; slices = 40; flip angle = 90°; FOV = 220 × 220 mm^2^; matrix size = 94 × 94; measurements = 150.

### 2.4. Rs-fMRI Preprocessing and Calculation

Imaging data preprocessing was performed in MATLAB R2015b using the toolbox for Data Processing and Analysis of Brain Imaging (DPABI, Yan et al. 2016, http://rfmri.org/DPABI, accessed on 3 September 2022) and Statistical Parametric Mapping (SPM) 12 (http://www.fil.ion.ucl.ac.uk/spm, accessed on 3 September 2022). The detailed preprocessing steps are as follows:

The first 10 time points of each subject were deleted to obtain reliable and stable data, leaving functional images for further analysis. Slice timing was used to correct the difference of slice acquisition times. These corrected images were then realigned to the first image to correct for head motion. All subjects with head movements in any direction causing a translation of 3 mm or a rotation of 3° were excluded. The remaining images were further co-registered to the T1 anatomical images for spatial normalization to the Montreal Neurological Institute (MNI) resampling, to 3 × 3 × 3 mm^3^ voxel size. The white matter signal, cerebral spinal fluid signal, and Friston 24 head motion parameters were regressed as nuisance covariates. Temporally bandpass filtering (0.01–0.1 Hz) was conducted to reduce low-frequency drift and physiological high-frequency noise, and linear trend removal was performed. Finally, the images were smoothed with a Gaussian kernel of 4 × 4 × 4 mm^3^ full-width at half maximum.

Homotopic resting-state FC was calculated as the Pearson correlation coefficient between each voxel’s residual time series and its symmetrical inter-hemispheric counterpart. The computed correlation coefficients were then converted into normalized z-map data using the Fisher Z-transformation. The resulting values were subsequently analyzed for VMHC alterations between groups.

Brain areas that showed significant group differences in VMHC maps were selected as seed regions to calculate FC. Seed-based FC analyses were performed using the functional images with all preprocessing steps completed. FC was defined as the correlation coefficients between the averaged time series of each seed and the time series of the remaining voxels in the whole brain. Fisher’s r-to-z transformation was applied in order to improve normality of the correlation coefficients.

### 2.5. Statistical Analysis

The statistical analyses of clinical data were conducted using SPSS version 22 (IBM Corporation, Armonk, NY, USA). Results were considered to be statistically significant at *p*-value < 0.05 for all statistical tests. The continuous variables of age and education level were compared between HCs and female MwoA patients using the two-sample *t*-test.

The imaging statistical tests were performed in the DPABI toolbox. The global VMHC values were calculated between HCs and MwoA patients using a two-sample *t*-test with gender, age and education level as the nuisance variables. The brain regions with significant differences in VMHC were defined as the mask; these mask VMHC values were extracted for further seed-based FC and correlation analysis. The statistical threshold was set at voxel level with *p* < 0.001 and cluster level with *p* < 0.05 for multiple comparisons using Gaussian random field (GRF) theory.

Pearson’s correlation analyses were performed between the VMHC and FC values in multiple regions, as well as the clinical scores for disease duration, migraine days, VAS, BAI and BDI for all MwoA patients. The significance level for these correlation analyses was set at *p* < 0.05.

## 3. Results

### 3.1. Baseline Demographic and Clinical Characteristics

After excluding subjects with motion artifact (*n* = 3) and incomplete clinical assessment (*n* = 1), the final groups were comprised of 43 female MwoA patients and 43 matched HCs. There were no statistically significant differences between the two groups for age or education level (*p* > 0.05) (Table 1).

### 3.2. VMHC Results

**Figure 2:**
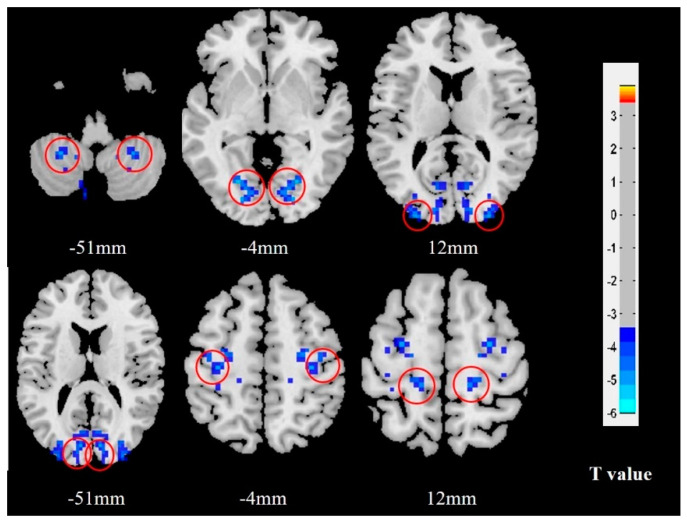
VMHC results in female MwoA patients relative to HCs. Brain regions with notable alterations in VMHC are indicated with red circles. The blue color represents deactivation of neural activity. MwoA patients demonstrated significantly decreased VMHC in the bilateral cerebellum, lingual gyrus, middle occipital gyrus, cuneus, precentral gyrus and postcentral gyrus compared with HCs. **Abbreviations:** VMHC, voxel-mirrored homotopic connectivity; MwoA, migraine without aura; HCs, healthy controls.

Compared with HCs, MwoA patients exhibited decreased VMHC in the bilateral cerebellum_8, lingual gyrus, middle occipital gyrus, cuneus, precentral gyrus and postcentral gyrus. There were no increases in VMHC in the brain regions of migraine patients with migraine compared with the HCs (Table 2 and Figure 2).

### 3.3. Seed-Based FC Results

**Figure 3:**
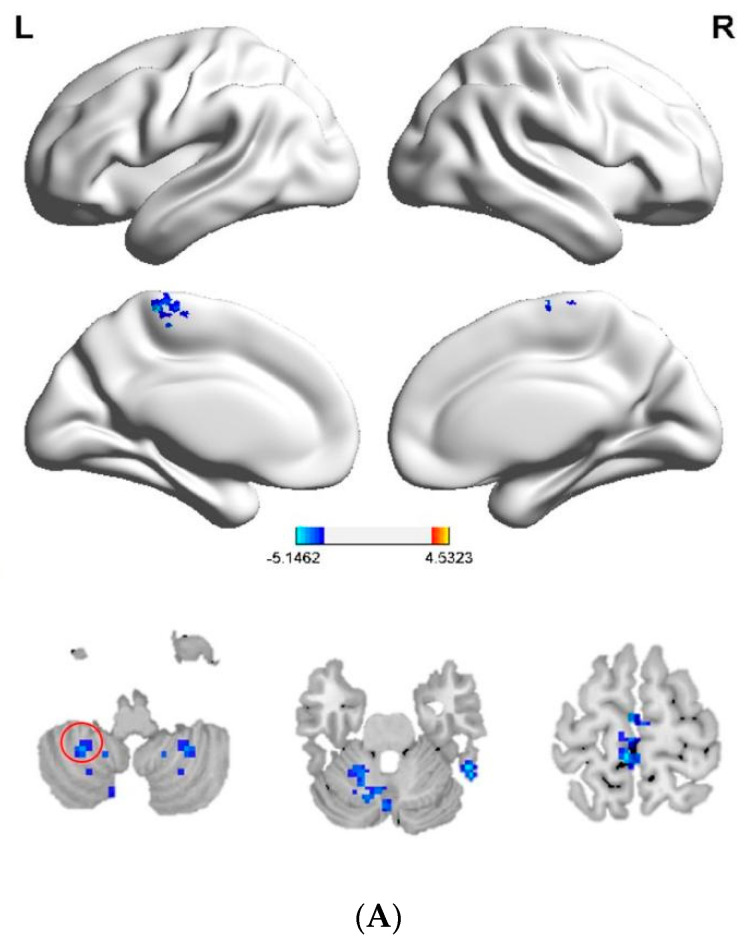

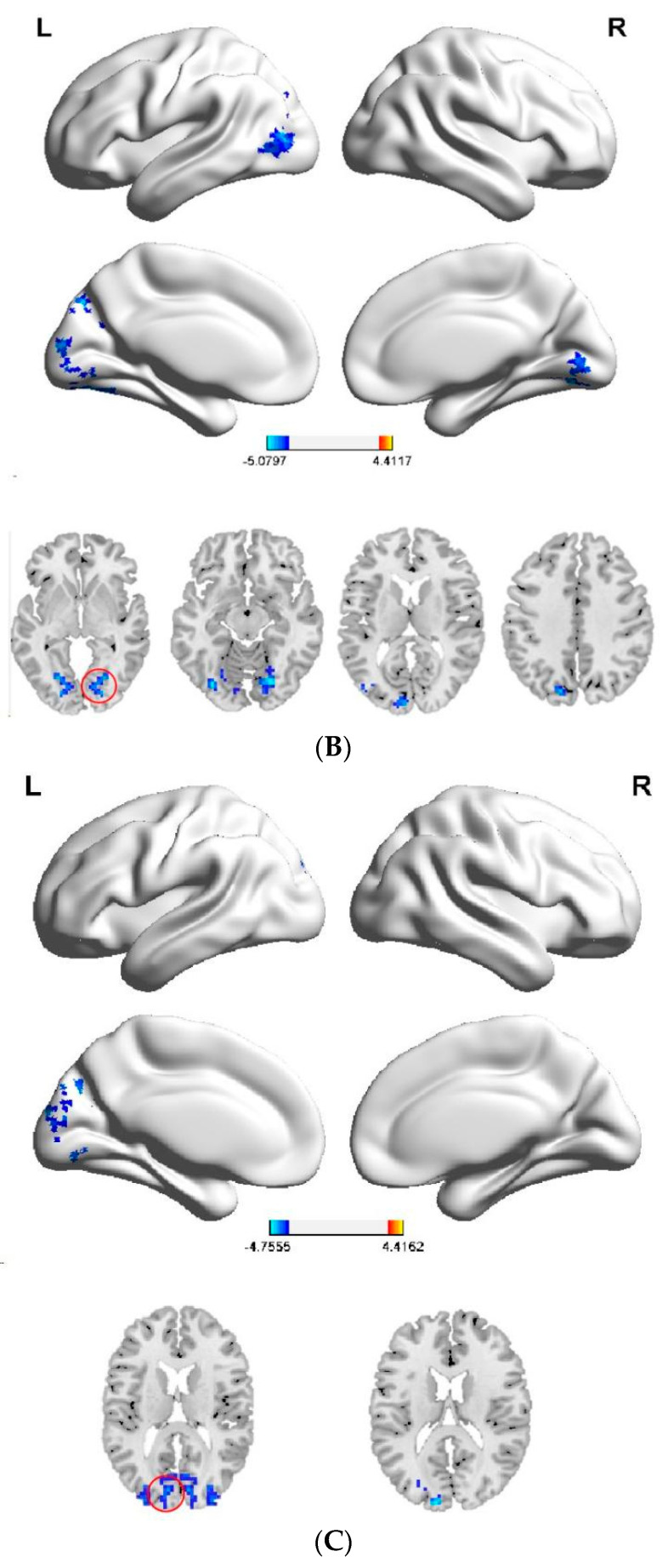

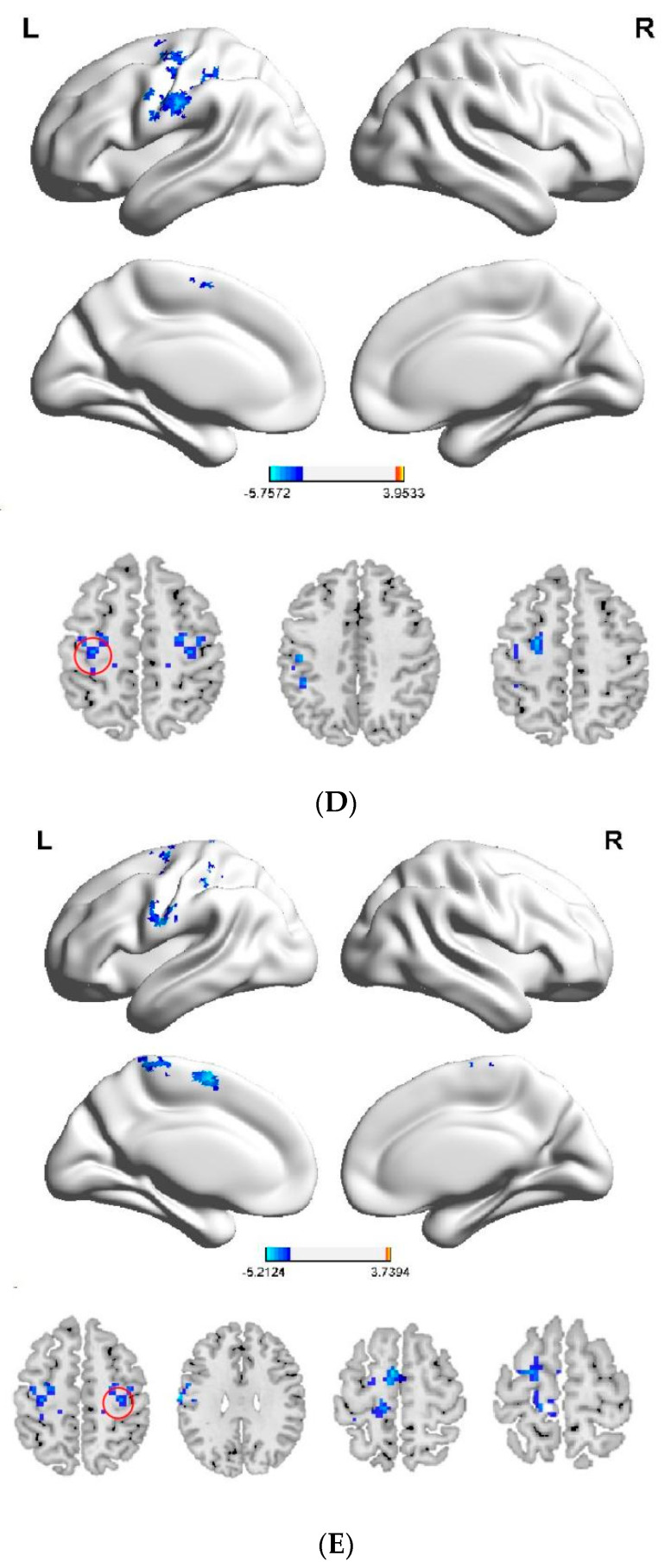

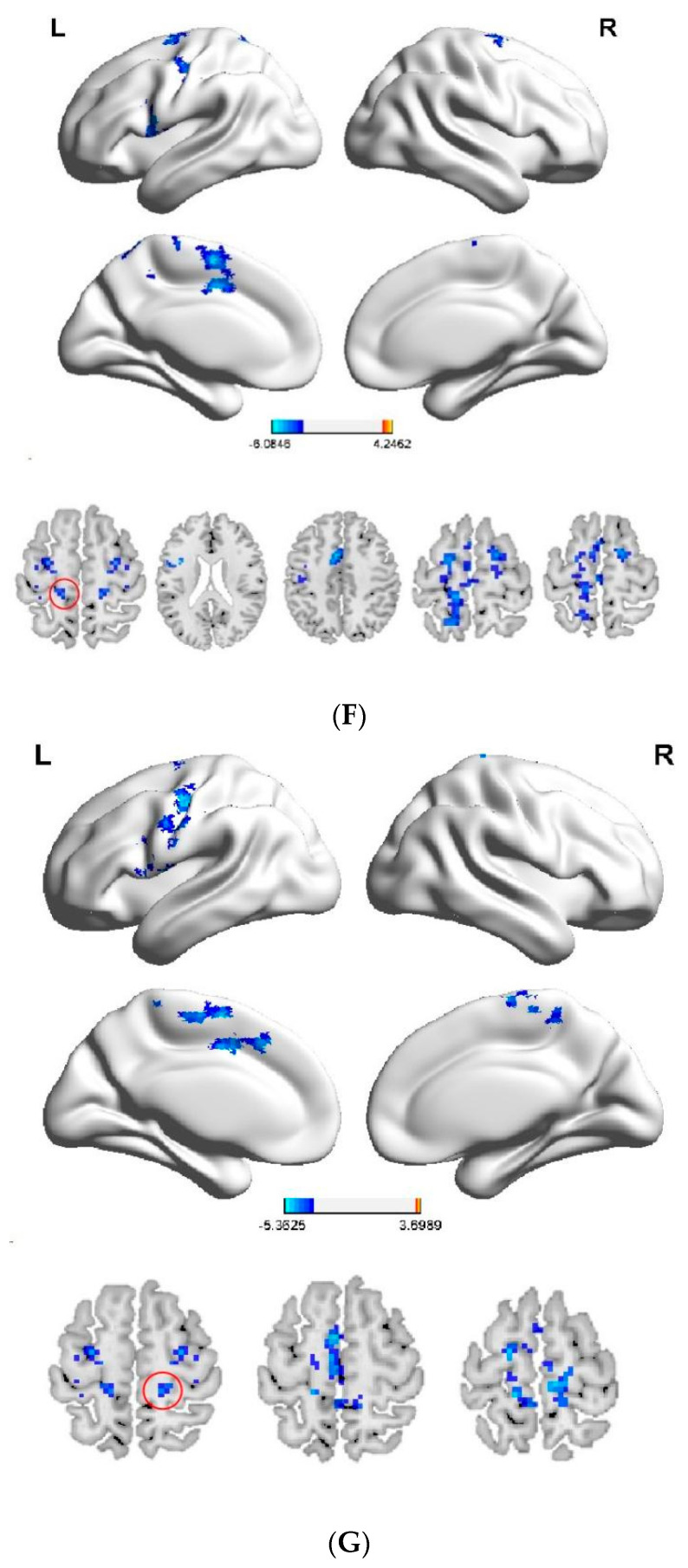
(**A**–**G**) Differences in seed-based FC between female MwoA patients and HCs. Red circles indicate seed regions. Blue colors represent decreased FC values. Relative to the HCs, patients exhibited decreased FC (**A**) between the right cerebellum_8 and both the right inferior temporal gyrus and left paracentral lobule; (**B**) between the left lingual gyrus and the left fusiform gyrus, left calcarine cortex, and left cuneus; (**C**) between the right cuneus and left superior occipital gyrus; (**D**) between the right precentral gyrus and both the left inferior parietal lobule and left superior frontal gyrus; (**E**) between the left precentral gyrus and the left postcentral gyrus, left supplementary motor area, and left paracentral lobule; (**F**) between the right postcentral gyrus and the left opercular part of inferior frontal gyrus, left median cingulate and paracingulate gyrus, left paracentral lobule, and right superior frontal gyrus; and (**G**) between the left postcentral gyrus and both the left supplementary motor area and left paracentral lobule. Abbreviations: FC, functional connectivity; MwoA, migraine without aura; HCs, healthy controls.

The seed-based FC analysis revealed that female MwoA patients had decreased FC between the right cerebellum_8 and both the right inferior temporal gyrus and left paracentral lobule; between the left lingual gyrus and the left fusiform gyrus, left calcarine cortex, and left cuneus; and between the right cuneus and the left superior occipital gyrus. Decreased FC was also identified between the right precentral gyrus and both the left inferior parietal lobule, and the left superior frontal gyrus; and between the left precentral gyrus and the left postcentral gyrus, the left supplementary motor area, and the left paracentral lobule. Additionally, FC was decreased between the right postcentral gyrus and the left opercular part of inferior frontal gyrus, the left median cingulate and paracingulate gyrus, the left paracentral lobule, and the right superior frontal gyrus; and between the left postcentral gyrus and both the left supplementary motor area and the left paracentral lobule (Table 3, Figure 3). No brain regions showed increased FC in the patients compared with the HCs.

### 3.4. Correlations between VMHC and FC in Multiple Brain Areas and Clinical Parameters

The VMHC value in the bilateral lingual gyrus negatively correlated with duration of disease, and positively correlated with migraine days. There was negative correlation between the VMHC value in the bilateral middle occipital gyrus and migraine days. The FC of the right cerebellum_8 and left paracentral lobule was negatively associated with VAS. In addition, FC of the left lingual gyrus and left calcarine cortex was negatively correlated with duration of disease and VAS. There was also a negative correlation between FC of the left precentral and left paracentral lobule with VAS (Table 4).

## 4. Discussion

In this study, we analyzed VMHC and seed-based FC alterations at resting state in female MwoA patients compared with HCs; furthermore, we identified correlations between neuroimaging data and clinical parameters from migraine patients. The rs-fMRI studies demonstrated that female MwoA patients had significantly decreased VMHC in the bilateral cerebellum_8, lingual gyrus, middle occipital gyrus, cuneus, precentral gyrus and postcentral gyrus compared with HCs. Our seed-based FC analysis indicated that MwoA patients exhibited remarkably disrupted intrinsic connectivity involving the cerebellum, DMN, visual network and SMN. Furthermore, the VMHC and FC abnormalities were negatively correlated with many clinical indices. These observed inter-hemispheric FC impairments provide an important new avenue to better understand the central mechanisms underlying migraine in women.

Migraine is affected by changes in sex hormones; for instance, perimenopause is associated with increased migraine, especially menstrual migraine. Furthermore, migraines related to menstrual episodes are considered more disabling and less responsive to treatment than non-menstrual episodes [28]. Recent brain fMRI studies supported the existence of functional differences between women and men, between migraine patients and HCs, and between female migraine patients with different hormone levels [6,29]. One study suggested that the FC impairments of the ACC were significantly correlated with pain severity, and pain-associated emotional disorders in menstrual migraine patients and, therefore, the ACC might be an important biomarker to differentiate migraines from HCs [30]. Additional research explored the difference in amplitude of low-frequency fluctuations and regional homogeneity between pure menstrual migraine and menstrually related migraine in the dorsolateral prefrontal cortex and medial prefrontal cortex [31]. Moreover, VMHC abnormalities have been observed in patients with menstrual migraine relative to matched HCs [23]. However, little is still known regarding VMHC changes in female migraine patents.

We conducted a group comparison of rs-fMRI data between female MwoA patients and HCs and observed impaired VMHC in some symmetrical brain regions and disrupted FC in different networks. Firstly, the cerebellum, which is a part of the pain matrix, contributes to diverse dimensions of pain processing associated with emotion, higher cognitive function, sensory function, and motor control [32,33]. Previous fMRI studies have demonstrated its functional and structural abnormalities in patients with migraine [34,35]. In an experiment on trigeminal nociception performed in 54 migraine patients and 54 matched HCs, the patients showed significantly increased grey matter volume in the cerebellar areas VI, VIIb, VIIIa, crus I, and crus II, along with increased activity between the PAG and left crus I (ipsilateral to the stimulation). The crus I was the same region functionally and structurally and was strongly associated with higher cognitive areas in the temporal, frontal, and parietal lobes of the cortex [36]. Another rs-fMRI study found enhanced FC of the right cerebellum with the ipsilateral medial prefrontal cortex, which may primarily participate in the pain perception through cognitive control mechanisms [37]. Both studies identified that the cerebellum was involved in migraine through its association with higher cognitive functions. Others have speculated that lobule VIIIB of the cerebellum is linked to secondary sensory processing in response to pain [38]. Our results demonstrated that female MwoA patients had weakened VMHC in the bilateral cerebellum and decreased FC between the right cerebellum_8 and certain pain-related regions. Negative correlations were observed between FC of both the right cerebellum_8 and left paracentral lobule with VAS, indicating that the cerebellum may participate in the processing of migraine onset and regulation.

Secondly, the DMN is the most commonly reported network associated with migraine [10] and includes regions such as the precuneus, dorsomedial prefrontal cortex, posterior cingulate cortex, thalamus, inferior parietal lobule, and inferior temporal cortex. A previous study conducted FC analysis of 20 MwoA patients and 20 HCs to identify changes in the DMN. Although they observed decreased FC in two core regions of DMN, namely, the prefrontal and temporal lobes, they did not find significant correlations between DMN dysfunction and brain structure or clinical and psychological indicators [39]. The frontal cortex is one of the most active areas of the brain in migraine sufferers. The prefrontal cortex plays a vital role in regulating pain perception through cognitive control mechanisms [40]. The temporal lobe is primarily responsible for language and auditory perception, and its activation has been shown to be involved in emotional responses to pain [41]. Our study found decreased FC in the frontal and temporal lobe along with many brain regions. Furthermore, impaired VMHC was found in the bilateral lingual gyrus, which belongs to the inferior parietal lobule that is involved in spatial discrimination and attention to pain [42]. We also observed decreased FC of the left inferior parietal lobule with the right precentral gyrus, left lingual gyrus, and left fusiform gyrus. Pearson correlation analysis showed that VMHC or FC of the lingual gyrus was positively correlated with VAS and negatively correlated with disease duration and migraine days.

Thirdly, the occipital lobe primarily helps with visual perception and processing. Previous studies suggested that structural and functional abnormalities of this region occur in migraine patients with aura [43], and these abnormal changes have also been observed in MwoA patients [44]. Recent research identified strong, positive dynamic connections within the visual cortex, as well as significant negative FC with the middle occipital gyrus and the posterior thalamus in MwoA patients; moreover, the negative connectivity of the two brain regions was significantly associated with migraine frequency [45]. In a related study, more than 75% (31/40) of MWoA patients experienced photophobia and/or phonophobia during migraine attacks. Therefore, the authors hypothesized that recurrent migraine attacks might be related to disturbances in the integration of visual and auditory signals caused by abnormal spontaneous activity in the occipital cortex [46]. Consistent with these previous studies, we also found a negative correlation between the VMHC value of bilateral middle occipital gyrus and migraine days. In addition, our study found decreased FC of the cuneus, calcarine cortex, and superior occipital gyrus with other regions. The cuneus, located on the occipital lobe, participates in visual selective attention by conveying top-down information from the attention network to the visual cortex [47]. The enhanced FC between the right thalamus and right cuneus has been shown in MwoA patients, and further correlation analysis demonstrated that FC changes between them were negatively related to generalized anxiety disorder scores [48]. The higher activation of the cuneus in the interictal period may be involved in a compensatory role in habituation deficits and headache relief. However, our finding of reduced VMHC within the bilateral cuneus was not in line with previous experiments, which may be explained by migraine-related disturbance in VMHC of the visual cortex in MwoA patients during resting state.

Lastly, the precentral gyrus, postcentral gyrus, and paracentral lobule together with the supplementary motor area constitute the SMN. Changes in the FC of this network and other cerebral cortexes affect the integration of multiple senses and the perception and processing of pain. Past research confirmed that MwoA patients had reduced FC of the SMN and other brain regions during the interictal period [49]. Disruption of FC in these regions may lead to dysfunction in visual processing, multisensory integration, nociceptive processing, spatial attention and intention, and cognitive assessment and regulation of pain. Recurrent headaches can lead to disruptions in the SMN. Pain sensitivity and patients’ quality of life are strongly associated with abnormal FC of the SMN with other brain regions. Recent research findings have increasingly validated the primary involvement of the postcentral gyrus in sensory-discriminative pain processing [50]. A previous seed-based rs-fMRI study associated with the brainstem revealed decreased FC between the right substantia nigra and the right postcentral gyrus, and that this hypo-connectivity between them was negatively correlated with migraine duration [51]. These disrupted networks may change the functional patterns of the somatosensory cortex, thereby resulting in the functional impairments seen in MwoA patients; furthermore, this disruption has also been found between the primary somatosensory cortex and temporal cortex [49]. Patients with menstrual migraine exhibited markedly increased VMHC of the bilateral postcentral gyrus compared with HCs [23]. Compared to males, female migraineurs showed increased PAG-FC with the somatosensory and motor cortexes [16]. The present study found decreased VMHC in the bilateral precentral gyrus and postcentral gyrus. Furthermore, in our subsequent correlation analysis between rs-fMRI data and clinical parameters, we observed negative associations with VAS and the left precentral and left paracentral lobule. Therefore, disruption of the inter-hemispheric FC in the SMN may be involved in the neural mechanism of migraine.

Although the present study revealed some significant findings, there are still several limitations. First, although decreased inter-hemispheric FC was identified in several brain regions and networks between patients and HCs, no increases in inter-hemispheric FC were found; therefore, the alterations we observed in VMHC and FC require further confirmation. Second, changes in hormone levels can affect the outcome of brain fMRI results; therefore, future explorations of rs-fMRI characteristics in female migraine patients should collect menstrual cycle information and hormone levels. Furthermore, rs-fMRI features of female migraine patients should be supplemented with follow-up studies during menstruation, perimenopause, and menopause. Third, the control group was comprised of healthy female subjects, and subsequent studies should match male migraine patients with migraine and male healthy volunteers. Finally, we have not clearly confirmed whether the functional alterations were in line with structural alterations in the brain; therefore, a multimodal analysis that contains both structural and functional data analysis should be conducted in future work.

## 5. Conclusions

We focused on the VMHC changes at resting state in female MwoA patients during the interictal period and observed that impaired inter-hemispheric FC predominately occurred in the cerebellum, DMN, visual network, and SMN in patients compared with HCs. These observed changes in FC in various brain networks were correlated with the duration, onset days, and severity of headache, which may be due to a decompensation in response to the headaches, and follow-up studies are needed to determine the causal relationship. Our results may help to better understand the central mechanism underlying female migraine onset and development, and provide an objective imaging marker for the identification of specific pathologic central targets in female migraine patients.

## Figures and Tables

**Table 1:** Demographics and clinical data of female MwoA patients and HCs (mean ± SD).

Parameter	MwoA Patients (*n* = 43)	HCs (*n* = 43)	*p* Value
Age (years)	38.19 ± 10.62	36.81 ± 11.65	0.570
Education level (years)	15.58 ± 2.64	15.91 ± 3.09	0.600
Duration of disease (years)	17.02 ± 9.58	NA	NA
Migraine days	6.84 ± 6.15	NA	NA
VAS	7.57 ± 1.42	NA	NA
BAI	9.95 ± 8.12	NA	NA
BDI	8.93 ± 6.16	NA	NA

Notes: The two-sample *t*-test was applied to compare age and education level between HCs and MwoA patients. A *p*-value < 0.05 was considered statistically significant. Abbreviations: MwoA, migraine without aura; HCs, healthy controls; VAS, visual analogue scale; BAI, Beck Anxiety Inventory; BDI, Beck Depression Inventory.

**Table 2:** Alterations in VMHC between MwoA patients and HCs.

AAL Brain Regions	Cluster Size	Coordinates in MNI (x, y, z)			Intensity
Cerebellum_8_R	52	27	−48	−48	−5.0155
Cerebellum_8_L	52	−27	−48	−48	−5.0155
Lingual gyrus_R	77	24	−69	−3	−5.2546
Lingual gyrus_L	77	−24	−69	−3	−5.2546
Middle occipital gyrus_R	45	30	−96	12	−5.0939
Middle occipital gyrus_L	44	−30	−96	12	−5.0939
Cuneus_R	97	9	−84	15	−4.747
Cuneus_L	97	−9	−84	15	−4.747
Precentral gyrus_R	99	33	−21	54	−4.8335
Precentral gyrus_L	99	−33	−21	54	−4.8335
Postcentral gyrus_R	37	15	−33	60	−4.7024
Postcentral gyrus_L	37	−15	−33	60	−4.7024

Abbreviations: VMHC, voxel-mirrored homotopic connectivity; MwoA, migraine without aura; HCs, healthy controls; AAL, Anatomical Automatic Labeling; MNI, Montreal Neurological Institute; R, right; L, left.

**Table 3:** Brain regions with decreased seed-based FC in MwoA patients relative to HCs.

Seed Regions	AAL Brain Regions (Right or Left)	Cluster Size	Coordinates in MNI (x, y, z)			Intensity
Cerebellum_8_R	Inferior temporal gyrus_R	38	54	−45	−27	−5.1462
	Paracentral lobule_L	67	−6	−33	66	−4.9339
Lingual gyrus_L	Fusiform gyrus_L	200	−33	−78	−12	−4.9866
	Calcarine cortex_L	70	−6	−93	12	−4.3339
	Cuneus_L	39	−15	−78	39	−4.713
Cuneus_R	Superior occipital gyrus_L	55	−12	−93	18	−4.7555
Precentral gyrus_R	Inferior parietal lobule_L	42	−42	−39	42	−4.7031
	Superior frontal gyrus_L	69	−24	−9	54	−5.1721
Precentral gyrus_L	Postcentral gyrus_L	53	−66	−12	30	−5.0832
	Supplementary motor area_L	88	−6	0	60	−5.2124
	Paracentral lobule_L	85	−12	−30	66	−4.9027
Postcentral gyrus_R	Opercular part of inferior frontal gyrus_L	104	−39	9	21	−5.8502
	Median cingulate and paracingulate gyrus_L	174	−6	6	45	−6.0579
	Paracentral lobule_L	157	−15	−33	66	−5.0365
	Superior frontal gyrus_R	57	18	−3	63	−5.1333
Postcentral gyrus_L	Supplementary motor area_L	148	−3	0	60	−5.3203
	Paracentral lobule_L	57	−18	−9	66	−4.8894

Abbreviations: FC, functional connectivity; MwoA, migraine without aura; HCs, healthy controls; AAL, Anatomical Automatic Labeling; MNI, Montreal Neurological Institute; R, right; L, left.

**Table 4:** Correlations between clinical parameters in female MwoA patients and VMHC and FC values in the abnormal bran regions and networks between female MwoA patients and HCs.

rs-fMRI	Brain Areas and Networks	Clinical Data	Correlation Coefficient	*p*-Value
VMHC	bilateral lingual gyrus	duration of disease	−0.352	0.021
		migraine days	0.317	0.039
	right middle occipital gyrus	migraine days	−0.374	0.013
	left middle occipital gyrus	migraine days	−0.380	0.012
FC	right cerebellum_8 and left paracentral lobule	VAS	−0.323	0.035
	left lingual gyrus and left calcarine cortex	duration of disease	−0.306	0.046
		VAS	−0.313	0.041
	left precentral and left paracentral lobule	VAS	−0.317	0.038

## Data Availability

The datasets analyzed or generated during the present study are available from the corresponding author on request.
